# Ethical implications of coronavirus disease 2019 for ENT surgeons – a discussion

**DOI:** 10.1017/S002221512000208X

**Published:** 2020-09-28

**Authors:** C G Leonard

**Affiliations:** Department of Otolaryngology – Head and Neck Surgery, Royal Victoria Hospital, Belfast, Northern Ireland, UK

**Keywords:** Ethics, Pandemics, Otolaryngology, Coronavirus

## Abstract

**Background:**

Coronavirus disease 2019 has had a dramatic effect on society and healthcare. Preparations were based on predictive models of need, and with uncertainty regarding risk to patients and healthcare workers. Actions taken had both immediate and ongoing ethical impacts. The most obvious of these was the shift in duty of care from individual patients to public health centred ethics and decision making.

**Relevance:**

In ENT, many procedures are aerosol-generating and so our capacity to provide care will remain significantly reduced. This reduction in capacity may result in difficult choices for patients when optimal care may be replaced by acceptable care. ENT surgeons may also be faced with unaccustomed paternalism when capacity prevents them from acting within the patients’ wishes.

**Conclusion:**

Despite these challenges, the novel uses of technology highlight the desire to preserve and enhance the autonomy of our patients.

## Introduction

As of 12th June 2020, there have globally been 7 390 702 confirmed cases and 417 731 deaths as a result of coronavirus disease 2019 (Covid-19), the condition caused by severe acute respiratory syndrome coronavirus-2 (SARS-CoV-2). In the UK alone, 290 147 people have been confirmed to have Covid-19, and 41 128 have succumbed to the condition.^1^ Furthermore, many numbers of healthcare workers have contracted the condition through their work, and we have sadly seen mortality within our own specialty.

Society as a whole and healthcare services in particular have had to prepare based on predictive models of need, and with uncertainty regarding risk to patients and healthcare workers themselves.^2^ Actions taken had both immediate and ongoing effects, changing every aspect of medical care overnight.^3^ Surgeons across all specialties saw elective work cancelled, and they were required to change both their model of work and the ethical principles of their day-to-day practice dramatically. We have all been faced, with and continue to face, issues relating to duty and standard of care, as well as our obligations to society, public health and ourselves.

## Discussion

The shift in duty of care from individual patients to public health centred ethics and decision making has been seen most dramatically in the cessation of elective surgery. The reduction in elective surgery freed up operating theatre space, ventilators and hospital beds for patients during a surge in Covid-19 cases, and preserved personal protective equipment (PPE) for those involved in their care. Delays in treatment on such a scale as this would not normally be tolerated, given the risk of harm both physical and psychological to individual patients. These concerns were overwhelmed by the moral need to save lives, prevent the spread of Covid-19 and ‘flatten the curve’.

As society undergoes a stepwise reduction in restrictions on liberty, so too healthcare must follow a stepwise path to recommence elective assessments and procedures. Those patients who stand to suffer most greatly from a delay or cancellation in their surgical procedure must be identified. Whilst it is perhaps not unusual to grade a waiting list by necessity, or to decide to cancel the patient last on the list as a result of an earlier delay, the scale of current restrictions is unfamiliar and shocking to most surgeons in the developed world. In the UK prior to Covid-19, we may have wished for more operating time, but now the use of the operating theatre and PPE is greatly restricted. In ENT, where many procedures are categorised as aerosol-generating procedures, operating theatre time may be at an even higher premium.^4^ As a result, the timely and evidenced-based care of even urgent conditions such as airway stenosis or malignancy is affected. The choices are not solely related to distributive justice or resource availability; we must also respect the autonomy of those patients requiring care. Whilst it would seem self-evident to treat a patient with cancer, we also know that such treatment will result in reduced World Health Organization performance status and increased vulnerability to the virus. Treatment requires exposure to the clinical setting, and the likelihood of iatrogenic infection in these high-risk patients, who are vulnerable to poorer outcomes, only enhances the difficulty of the choices we must support patients in making.^5^

The immediate changes in care during the Covid-19 peak also resulted in a dramatic shift in the day-to-day work of ENT surgeons. Remote access to in-patient data and imaging, as well as telemedicine clinics, allowed for the remote provision of guidance and surveillance of patients. These methods enabled the triage of those patients requiring management by face-to-face services. Having the minimum staff required in the hospital, whilst contrary to the routine and lifestyle of many surgeons, served the important purpose of preserving the medical workforce so as to prevent shortage at a later date.

These changes in practice draw into focus the standard of care provided, as Covid-19 shifted the boundaries of what is optimal treatment and what is acceptable treatment. This challenging ethical issue is not new to ENT given the breadth of clinical practice provided in our specialty. An otologist must be trained in and be capable of draining a deep space neck infection, just as a head and neck surgeon must be capable of a cortical mastoidectomy. This necessity is reflected throughout ENT training.^6^ In light of the potential numbers of patients with Covid-19, many ENT surgeons found themselves called upon to function outside their normal zone of practice. Whilst undoubtedly challenging, both morally and clinically, for the individuals working in Nightingale units or elsewhere, ENT surgeons have a breadth of skills and ability to help patients beyond the narrow confines of their subspecialties. The application of these skills upholds our duty and responsibility as doctors to care, and is in keeping with the history of both our specialty and our profession.

The ongoing requirement to find balance between what is optimal and what is acceptable makes a shift to greater paternalism more likely than we have been used to in recent practice. Surgical care in the post-Montgomery era (Montgomery *vs* Lanarkshire Health Board case (2015), which concerned risk disclosure for informed consent), and the General Medical Council guidance entitled ‘*Consent: Patients and Doctors Making Decisions Together*’, is focused on information provision, discussion, respect for patient values and shared decision making.^7,8^ The restrictions placed on out-patient and operative space discussed above may increasingly result in an inability to respect our patients’ autonomy and right to self-determination. Initially, it is important to clarify with patients if they are willing to accept the risk of undergoing surgery during the Covid-19 pandemic. In light of the Montgomery ruling, this will require discussion of the screening process in place for both operating theatre staff and the patient themselves. It does, however, seem likely that as numbers of new referrals continue to outstrip patients completing surgical care, the frequency with which we are able to respect patient choice in a timely fashion may fall below both what has happened historically and what would be deemed either optimal or acceptable.

The scarcity of PPE, and the potential for shortages both during the initial peak in cases and going forward, draws the responsibility and duty of care for all clinicians into further consideration. The duty of care to work in environments outside of our norms is clear; however, that responsibility does not extend to working in those environments without adequate PPE. During the initial Covid-19 peak in the UK and abroad, illness within healthcare was a significant concern; as such, medical and allied health staff became a limited resource. To practice in the absence of adequate PPE is not so much an act of heroism as an irresponsible act that may reduce the efficacy of care.

## Conclusion

Despite the shift in duty of care from individual patients to public health centred ethics, the impact on care provision and decision making, and the potential increase in paternalism, the Covid-19 pandemic reminds us all of the need to continue to meet the moral premise of all medical practice, to help our patients. The efforts around the country, and indeed the world, to reduce patient waiting times and risk, through novel uses of telemedicine, remote communication with colleagues, enhanced triage of referrals and many other means, highlight the desire to preserve and enhance the autonomy of our patients.
